# Increased Transglutaminase 2 Expression and Activity in Rodent Models of Obesity/Metabolic Syndrome and Aging

**DOI:** 10.3389/fphys.2020.560019

**Published:** 2020-09-15

**Authors:** Krishna C. Penumatsa, Ines Falcão-Pires, Sara Leite, Adelino Leite-Moreira, Chinmayee D. Bhedi, Sabina Nasirova, Jing Ma, Roy L. Sutliff, Barry L. Fanburg

**Affiliations:** ^1^Pulmonary Critical Care and Sleep Division, Department of Medicine, Tufts Medical Center, Boston, MA, United States; ^2^Faculty of Medicine of the University of Porto, Cardiovascular Research and Development Center, Porto, Portugal; ^3^Department of Medicine, Atlanta Veterans Affairs and Emory University Medical Centers, Atlanta, GA, United States; ^4^Department of Medicine, Emory University, Atlanta, GA, United States

**Keywords:** transglutaminase 2, heart, lung, obesity, metabolic syndrome, aging, tissue stiffness, diastolic dysfunction

## Abstract

Diastolic dysfunction of the heart and decreased compliance of the vasculature and lungs (i.e., increased organ tissue stiffness) are known features of obesity and the metabolic syndrome. Similarly, cardiac diastolic dysfunction is associated with aging. Elevation of the enzyme transglutaminase 2 (TG2) leads to protein cross-linking and enhanced collagen synthesis and participates as a candidate pathway for development of tissue stiffness. With these observations in mind we hypothesized that TG2 may be elevated in tissues of a rat model of obesity/metabolic syndrome (the ZSF 1 rat) and a mouse model of aging, i.e., the senescent SAMP8 mouse. In the experiments reported here, TG2 expression and activity were found for the first time to be spontaneously elevated in organs from both the ZSF1 rat and the SAMP8 mouse. These observations are consistent with a hypothesis that a TG2-related pathway may participate in the known tissue stiffness associated with cardiac diastolic dysfunction in these two rodent models. The potential TG2 pathway needs better correlation with physiologic dysfunction and may eventually provide novel therapeutic insights to improve tissue compliance.

## Introduction

It is well known that the pathophysiology and progression of multiple human diseases are associated with tissue stiffness (or loss of compliance). Although such disease processes may also involve other non-contractile organs such as the kidney and liver, they are most evident in contractile tissues such as heart, lungs, and the vasculature. The stiffness is thought to be largely derived from changes in tissue extracellular matrix that consists of proteins such as fibronectin, collagen, and elastin. While stiffness of heart tissue may result in congestive heart failure, that of the lungs may produce respiratory failure and that of the circulation increases risk of hypertension.

The multifunctional enzyme transglutaminase 2 (TG2) is well known to post-translationally modify proteins by either creating cross-links between proteins at glycine and lysine residues (Greenberg et al., 1991; Griffin et al., 2002) or by the proteins with primary amine groups (Penumatsa and Fanburg, 2014; Muma and Mi, 2015; Lai and Lin, 2017). The food industry utilizes bacterial transglutaminase to enhance palatability of meats by a stiffening process (Kieliszek and Misiewicz, 2014). There has been a gradual accumulation of data that associates TG2 with synthesis and cross-linking of extracellular matrix proteins that produces tissue stiffness (Mosher and Schad, 1979; Orban et al., 2004). Highlighting the critical role of TG2 in tissue remodeling, recent studies by Santhanam et al. demonstrated that TG2 mediates ventricular (Oh et al., 2017) and aortic (Steppan et al., 2017) contractility, stiffness and function. Furthermore, the events of TG2 alterations are intricately interwoven with influences on transcription factors (Kumar and Mehta, 2012; Brown, 2013; Penumatsa et al., 2014), inflammation (Liu et al., 2019), oxidative stress (Lee et al., 2019), and fibrogenic signaling (Olsen et al., 2014; Penumatsa et al., 2017). Thus, TG2 is a potential target for disruption of tissue stiffness that occurs in diseases of the cardiovascular system and lungs.

In the present study, TG2 expression and activity were measured by immunoblotting in rodent models of obesity/metabolic syndrome and aging. Serotonylation of fibronectin (sFn), a technique previously developed by our laboratory (Liu et al., 2011), was used as a surrogate marker for increased TG2 transamidation activity. Our data indicate for the first-time that metabolic dysfunction and aging are associated with increased TG2 expression and activity in multiple tissues, including heart and lungs.

## Materials and Methods

### Animal Models

Male Wistar Kyoto (control; *n* = 6), ZSF1 lean (*n* = 6), and obese (*n* = 6) rats were obtained from Charles River (Barcelona, Spain) and fed with Purina Diet (#5008). By their 18th week of age, rats underwent metabolic, aerobic capacity, and echocardiographic evaluation. One to two weeks later, the rats were deeply anesthetized with 8% sevoflurane and immediately euthanized by exsanguination. Tissue samples were collected for molecular studies, as described below. All rat studies followed the recommendation of the Guide for the Care and Use of Laboratory Animals, published by the US National Institutes of Health (NIH Publication No. 85–23, Revised 2011) and were certified by the Portuguese Veterinary Governmental Authorities, approved by the Portuguese Foundation for Science and Technology and by the ethical committee of the institution. Only trained researchers, certified with a Laboratory Animal Science course according to the Federation of European Laboratory Animal Science Associations, performed rat handling and procedures. Aging mouse models, senescence-accelerated prone mice (SAMP8; *n* = 6) and senescence-accelerated resistant mice (SAMR1; control; *n* = 6) were purchased from Envigo (Formerly Harlan) (Indianapolis, IN, United States). All mouse studies were carried out using male 30-week-old mice fed *ad libitum* with irradiated 18% protein global rodent diet (Teklad 2918) (Reed et al., 2011). All mouse studies were performed in accordance with protocols approved by the Atlanta Veterans Affairs Institutional Animal Care and Use Committee.

### Aerobic Capacity and Exercise Tolerance

On week before euthanasia, the rats were placed on a treadmill chamber coupled to a gas analyzer (LE8700C and LE405, Panlab Harvard Apparatus^®^). The gas flow was set at 700 mL.min^–1^ and treadmill was tilted at 10°. The adaptation was carried out at a speed of 15 cm.s^–1^ for 3 min. The maximum stress test started at a speed of 30 cm.s^–1^, with increments of 5 cm.s^–1^ every minute until the rats reached maximal aerobic capacity (VO_2_ max).

### Metabolic Studies

After 12 h of fasting, rats underwent glycemic measurements (Freestyle-Mini) at baseline and after 15, 30, 60, 90, and 120 min of 1 g.Kg^–1^ glucose gavage (oral glucose tolerance test) or a 0.5 U.Kg^–1^ intraperitoneal insulin injection (insulin resistance test).

### Echocardiographic Evaluation

Anesthetized rats (3–4% sevoflurane) were placed on a heating pad and the ECG was monitored. A linear 15 MHz probe (Sequoia 15L8W) was used and a parasternal short axis view was performed to record M-mode as previously described (Hamdani et al., 2013). Aortic and mitral flow tracing velocity were recorded by pulsed-wave Doppler. Peak systolic tissue velocity and E’ were measured with tissue Doppler at the medial mitral annulus. Maximal left atrial dimensions were measured by 2D echocardiography in a four-chamber view. Acquisitions were done while transiently suspending mechanical ventilation and recordings from three consecutive heartbeats were averaged.

### Western Blot Analysis

Tissue samples of both rats and mice were minced and homogenized in NP-40 lysis buffer (Boston Bio Products) containing protease and phosphatase inhibitor cocktails (MilliporeSigma). Tissue homogenates were centrifuged, and the protein content was determined using a Bradford Assay (Bio-Rad Laboratories). Reduced samples were then subjected to SDS-PAGE as described previously (Liu et al., 2011; Penumatsa et al., 2014). Western blotting analysis of total protein lysates were performed using the following primary antibodies: serotonin (5-HT; Sigma, Cat# S5545); transglutaminase 2 (TG2; Santa Cruz Biotechnology, Cat# sc-48387); β-actin (Cell Signaling Technology, Cat# 4970) and GAPDH (Cell Signaling Technology). Horseradish peroxidase-conjugated secondary antibodies (Santa Cruz Biotechnology) were used for immunoreactive protein detection. Serotonylation of fibronectin (sFN) represents TG2 transamidation activity (Liu et al., 2011). An ECL Western blotting substrate (Thermo Fisher Scientific) was used to detect the protein bands. Densitometry analysis was performed using Un-Scan-It gel analysis software (Silk Scientific, Orem, UT) as previously described (Penumatsa et al., 2014). The band intensity of the target protein was normalized against the β-Actin or GAPDH bands and the data was expressed as fold change in expression compared to respective controls.

### Statistical Analysis

Data were expressed as mean ratios ± standard error of mean (SEM). Student’s *t*-test or One-way analysis of variance (ANOVA) followed by Tukey’s *post hoc* test (SigmaPlot 12.5 software; Systat Software, San Jose, CA, United States) were used to compare experimental groups. A *p* < 0.05 was considered statistically significant.

## Results

### TG2 Expression and Activity Are Increased in ZSF1 Obese Rats

Consistent with our previous studies (Hamdani et al., 2013), obese rats presented higher body, heart and peri-renal fat weight than lean ZSF1 and Wistar-Kyoto rats (Table 1). In addition, Obese ZSF1 rats showed hyperglycemia, impaired glucose tolerance and insulin resistance, consistent with the diagnosis of metabolic syndrome. Obese rats presented signs of congestion of the lungs, as assessed by increased weight and effort intolerance (lower values of VO_2_ max). Moreover, Table 1 displays other features that indicate a phenotype of HFpEF in ZSF1 obese rats (Leite et al., 2015a,b; van Dijk et al., 2016) and not in ZSF1 lean or Wistar Kyoto rats, such as normal left ventricular systolic function, as observed by similar cardiac index (CI) and ejection fraction (EF), and signs of diastolic dysfunction (increased E/e’ and left atrium area).

**TABLE 1:** Hemodynamic and metabolic changes in ZSF1 obese rats.

	**Wistar Kyoto (*n* = 6)**	**ZSF1 lean (*n* = 6)**	**ZSF1 obese (*n* = 6)**
**Morphometry**			
Body weight, g	347 ± 8	454 ± 21*	599 ± 7*^†^
TL, mm	37.7 ± 0.4	40.7 ± 0.5*	40.8 ± 1.4*
Heart weight/TL, mg/mm	33.5 ± 1.2	37.5 ± 2.1	41.4 ± 2.1*
Lung weight/TL, mg/mm	33.7 ± 1.9	41.8 ± 1.6*	48.6 ± 2.6*
Perirenal fat/TL, mg/mm	65.8 ± 4.6	85.3 ± 12.7	348.5 ± 24.7*^†^

**Metabolic studies**			
Fasting glucose, mg/dl	82.5 ± 8.4	75.7 ± 3.6	168.2 ± 14.2*^†^
Glucose AUC_OGTT_, mg/dl/h	203 ± 9	192 ± 12	500 ± 36*^†^
Glucose AUC_ITT_, mg/dl/h	109 ± 4	104 ± 6	329 ± 23*^†^

**Effort test**			
O_2_max, ml/min/cm^2^	38.5 ± 1.5	39.4 ± 0.9	22.7 ± 1.5*^†^

**Echocardiography**			
CI, μl/min/cm^2^	228 ± 15	330 ± 47	352 ± 51
EF,%	79 ± 4	81 ± 3	82 ± 3
LAA_i_, cm^2^/m^2^	4.9 ± 0.2	5.0 ± 0.2	6.5 ± 0.6*^†^
E/e 12.4^±^ 1.0	14.2 ± 0.5	16.9 ± 1.1*

AUC, area under curve; CI, cardiac index; EF, ejection fraction; E/e^±^ SEM, n = 6 rats per group. *P < 0.05 vs. Wistar Kyoto rats, ^†^P < 0.05 vs. ZSF1 Lean rats by one-way ANOVA.

**FIGURE 1:**
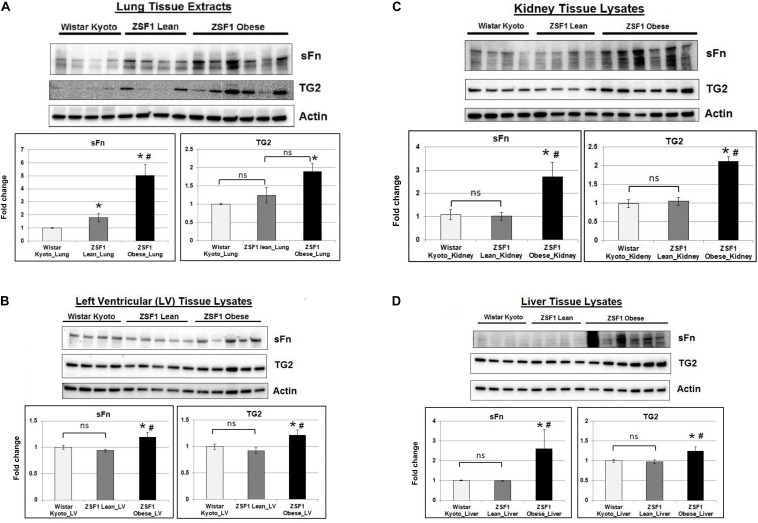
TG2 activity and expression are increased in a rat model of metabolic syndrome. Representative Western blot images showing serotonylated fibronectin (sFn) and transglutaminase 2 (TG2) expression levels in **(A)** lung, **(B)** cardiac left ventricular, **(C)** kidney, and **(D)** liver tissues of Wistar Kyoto, ZSF1 lean and ZSF1 obese rats. Beta-actin was used as a loading control. Bar graphs showing the normalized fold change differences compared to Wistar Kyoto rats. Data are expressed as mean ± SEM. *n* ≥ 4 rats per group. *p < 0.05 compared to Wistar Kyoto rats. ^#^*p* < 0.05 compared to ZSF1 lean rats by one-way ANOVA. ns = not significant.

Given that the ZSF1 obese rats show signs of metabolic dysfunction, we then assessed the TG2 expression and activity in multiple tissues. TG2 expression was significantly increased in the lungs (Figure 1A) of ZSF1 obese rats compared to control Wistar Kyoto rats. Additionally, we found increased TG2 transamidation activity as measured by sFn in lung tissues of both lean and obese ZSF1 rats compared to Wistar Kyoto rats (Figure 1A). Consistent with the observation that the ZSF1 obese rats show left ventricular diastolic dysfunction, both TG2 expression and activity were significantly increased in the left ventricles (Figure 1B). Consistent with the diabetic nephropathy observed in ZSF1 obese rats, TG2 levels are increased in kidneys (Figure 1C) and livers (Figure 1D) of this rat model.

### TG2 Expression and Activity Are Increased in SAMP8 Mice

**FIGURE 2:**
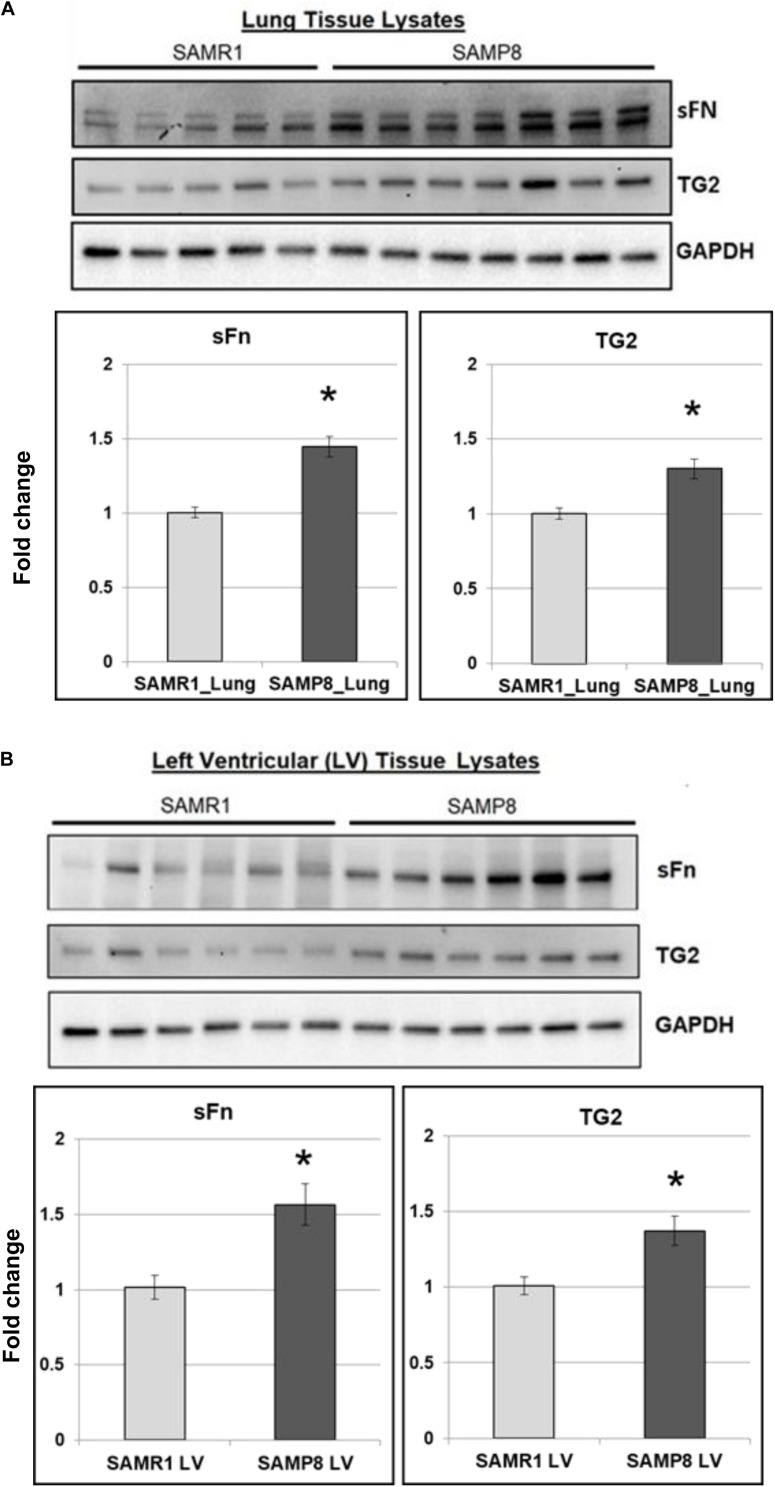
TG2 activity and expression and fibrogenic markers are increased in mouse model of aging. Representative Western blot images showing serotonylated fibronectin (sFn) and transglutaminase 2 (TG2) expression levels in **(A)** lungs and **(B)** left ventricles of senescence-resistant (SAMR1) and senescence-accelerated (SAMP8) mice. GAPDH was used as a loading control. Bar graphs showing the normalized fold change differences compared to SAMR1 mice. Data are expressed as mean ± SEM. *n* ≥ 5 mice per group. *p < 0.05 compared to SAMR1 mice by *t*-test.

The senescence-accelerated prone mouse (SAMP8) model of aging demonstrates several hallmarks of human cardiovascular pathology including aging-associated oxidative stress (Gan et al., 2012), metabolic dysfunction (Kurokawa et al., 1998), inflammation (Forman et al., 2011) and HFpEF (Reed et al., 2011). To determine the impact of aging on pro-fibrogenic phenotype, TG2 expression and activity were measured by Western blot analysis. We report here for the first-time that TG2 expression and activity are increased in lungs (Figure 2A) and left ventricles (Figure 2B) of senescent-prone (SAMP8) mice compared to control senescent-resistant (SAMR1) mice.

## Discussion

Our present studies establish that expression and activity of TG2, a protein cross- linking enzyme with fibrogenic properties, are increased in multiple organs from rodent models of both metabolic dysfunction and senescence. Additionally, we have reported previously that hypoxia (Penumatsa et al., 2017) and hyperglycemia (Bhedi et al., 2020) stimulate expression and activity of TG2 in cardiac and pulmonary vascular fibroblasts in culture. Although we have not yet specifically related TG2 activity to tissue compliance, our present *in vivo* observations with rodent models of metabolic syndrome (ZSF1 obese rats) and aging (SAMP8 mice) are consistent with participation of TG2 in cardiac and lung matrix changes that are associated with tissue stiffness in multiple cardiovascular and pulmonary diseases mediated by these conditions.

### TG2 in Pressure Overload Induced Cardiopulmonary Remodeling

The multifunctional enzyme TG2 is now receiving attention for its potential role in vascular (Bakker et al., 2006; Penumatsa et al., 2014; Steppan et al., 2014), cardiac (Kashima et al., 1997; Small et al., 1999; Wang et al., 2018), and pulmonary (DiRaimondo et al., 2014; Olsen et al., 2014) diseases. Our previous studies have identified that TG2 activity is largely controlled by physiologic events associated with increased cardiac pressure overload models, in particular those of chronic hypoxia (DiRaimondo et al., 2014; Penumatsa et al., 2017) and transverse aortic constriction (Bhedi et al., 2020). Studies by others have largely supported this concept (Baandrup et al., 2011; Shinde et al., 2018; Wang et al., 2018). A particular phenotype of cardiac disease characterized by increased stiffness or impaired relaxation is that of diastolic dysfunction. When combined with signs and symptoms of heart failure and a normal systolic function, diastolic dysfunction leads to heart failure with preserved ejection fraction (HFpEF) (Kass et al., 2004; Piccini et al., 2004; Reed et al., 2011). In addition, a possible common denominator of these events is that TG2 activity is under regulation by glycolysis and perhaps specifically the terminal glycolytic enzyme phosphokinase M2 (Bhedi et al., 2020).

### TG2 in Hyperglycemia Mediated Cardiopulmonary Remodeling

There is now general recognition that the pathology of diabetes mellitus (DM) includes remodeling of extracellular tissue and intercellular matrices with the formation of fibrosis and collagen alterations that promote tissue stiffness (Ban and Twigg, 2008; Law et al., 2012). Although fibrosis may be present in any organ of the body (Gilbert and Cooper, 1999; Patil et al., 2011), its involvement of the cardiovascular system is particularly prevalent in DM. While there may be more subtle processes involving the pulmonary arterial stiffness and cardiac right ventricle in association with pulmonary hypertension (PH) in DM (Grinnan et al., 2016; Whitaker et al., 2018), metabolic dysfunction has been identified in particular (Irwin et al., 2014; Lai et al., 2019). Up to 30% of patients with HFpEF have DM (Piccini et al., 2004). The basic mechanisms by which the intercellular matrix alteration occurs in DM are receiving increased attention. Appealing theories regarding mechanisms include direct effects of hyperglycemia on fibroblasts to promote their transition to myofibroblasts and subsequent fibrosis; glycation of proteins to form advanced glycation end products (AGEs) that alter the tissue matrices; and increased oxidative stress and inflammation (Asbun and Villarreal, 2006; Ban and Twigg, 2008). Studies have demonstrated associations between hyperglycemia (Skill et al., 2004; Bhedi et al., 2020), reactive oxygen species (Lee et al., 2019) and inflammatory (Liu et al., 2019) responses and TG2 regulation. Of interest, TG2 -mediated protein serotonylation has been found to be modulated in pancreatic beta cells (Paulmann et al., 2009).

We have proposed that TG2 is an important mediator of an alteration in intercellular matrix protein cross-linking that occurs in DM. We have tested a mouse model of type 2 diabetes, the Akita mouse, for expression and activity of TG2 in multiple organs and have identified an elevation of TG2 expression and activity in all organs studied as compared to controls (Bhedi et al., 2020). These observations further led us to examine the response of fibroblasts in culture to elevated glucose. We reported that in addition to stimulation of TG2, glucose elevates glycolysis, synthesis of TGFβ1 and cellular collagen content (Bhedi et al., 2020). In these studies we found that elevated synthesis of TGFβ1, type 1 collagen and α-smooth muscle actin are blocked by inhibition of TG2 or glycolysis (Bhedi et al., 2020). These observations suggest that enhanced glycolysis and TG2 production induced by hyperglycemia may lead to enhanced tissue stiffness either as a result of fibrosis or alteration of cardiac contractile mechanisms influenced by TG2.

### TG2 in Obesity/Metabolic Syndrome Mediated Cardiopulmonary Remodeling

It is well known that alterations in tissue matrix reflected as fibrosis or other alterations resulting in increased tissue stiffness occur in obesity and specifically in obesity associated with metabolic syndrome (Cavalera et al., 2014; Aroor et al., 2019). These features of the disease manifest as HFpEF and loss of pulmonary and systemic arterial compliance (Lai et al., 2019; Leite et al., 2019). Although animal models of the metabolic syndrome, such as the one used in the present study, have been identified as having increased tissue stiffness, the levels of TG2 activity in tissues of these animal models have never been evaluated. The present study was designed to address this deficiency. We have utilized ZSF1 rats with obesity, diabetes and hypertension as a model of the metabolic syndrome. This model has been used previously by Hamdani et al. for studies in which they demonstrated the presence of left ventricular diastolic dysfunction (Hamdani et al., 2013). With this model, we have now found for the first-time elevation of both TG2 expression and activity in multiple tissues including heart, kidney and liver of ZSF1 obese rats compared to ZSF1 lean and control ones. These results suggest a more generalized elevation of tissue TG2 in these animals, perhaps attributed to a hyperglycemia and/or an inflammatory phenotype. Interestingly, TG2 was elevated in lungs of both lean and obese ZSF1 rats compared control rats. Given that both lean and obese ZSF1 rats have previously shown elevated systemic blood pressure (Griffin et al., 2007), further studies are needed to assess the pulmonary circulation in these rat models.

### TG2 in Aging Mediated Cardiopulmonary Remodeling

Congestive heart failure is often associated with cardiac fibrosis and HFpEF (Wong et al., 1989; Biernacka and Frangogiannis, 2011; Loffredo et al., 2014; Diez-Villanueva and Alfonso, 2016). Clinical prevalence of HFpEF increases with aging (Fleg and Strait, 2012; Strait and Lakatta, 2012; Loffredo et al., 2014). Aging-associated fibrosis also occurs in other organs, including lungs (Schafer et al., 2017; Sicard et al., 2018); the prevalence of chronic lung diseases and idiopathic pulmonary fibrosis in the elderly may reflect this occurrence (Budinger et al., 2017). It has been previously reported that TG2 mediates cardiac ventricular (Oh et al., 2017) and systemic arterial (Armstrong et al., 2018) stiffness in rodent models of aging. Similar to that of the obesity/metabolic syndrome, it has been proposed that the aging process may be under the regulation of enhanced glycolysis with reduced mitochondrial function (James et al., 2015; Feng et al., 2016; Cho et al., 2017; Jang et al., 2018). Of note, altered glucose metabolism (Costantino et al., 2016; Cho et al., 2017; Zank et al., 2018) and inflammation (including elevation of cytokines) (Maggio et al., 2006; Forman et al., 2011) have been recognized to be driving metabolic forces in aging-associated tissue dysfunction.

Our current findings extend previous observations indicating an elevation of TG2 expression and activity in hearts and lungs of mouse models that experience glycolysis-inducing events such as exposure to hypoxia and transverse aortic banding which imposes a pressure overload on the left ventricle (Bhedi et al., 2020). Further studies are needed to determine the mechanisms that promote increased TG2 in these animal models and to relate these findings to the physiologic process of decreased tissue compliance. Since small molecule inhibitors of TG2 are currently available (Kim, 2018) and anti-TG2 monoclonal antibodies are being developed, these novel therapeutics may prove to be useful in modifying tissue stiffness occurring with alterations in tissue pressure-overload, diabetes, obesity/metabolic syndrome or aging.

## Data Availability Statement

All datasets generated for this study are included in the article/supplementary material.

## Ethics Statement

The animal studies were reviewed and approved by the Portuguese Foundation for Science and Technology or Atlanta VA IACUC.

## Author Contributions

KP, IF-P, RS, and BF conceived and designed the experiments and wrote the manuscript. KP, IF-P, SL, AL-M, CB, SN, JM, and RS performed the experiments. KP, IF-P, SL, AL-M, CB, and SN analyzed the data. All authors contributed to the final manuscript.

## Conflict of Interest

The authors declare that the research was conducted in the absence of any commercial or financial relationships that could be construed as a potential conflict of interest.
